# Do They Practice What They Preach? A Cross-Sectional Study of Heart-Healthy Dietary, Exercise, and Sleep Habits in US Medical Students

**DOI:** 10.1007/s40670-026-02718-3

**Published:** 2026-04-11

**Authors:** Harris Ziggy Whiteson, Madison Drogy, David Haner Wasserstein, Jane Qingyi Chen, Jay Ayar, Elizabeth Drugge, Kathryn Spanknebel, William H. Frishman, Kristina H. Petersen

**Affiliations:** 1 School of Medicine, New York Medical College, 40 Sunshine Cottage Rd, Valhalla, NY 10595 USA; 2 Department of Public Health, School of Health Sciences and Practice, New York Medical College, Valhalla, NY USA; 3 Department of Surgery, School of Medicine, New York Medical College, Valhalla, NY USA; 4 Department of Medicine, School of Medicine, New York Medical College, Valhalla, NY USA; 5 Department of Biochemistry & Molecular Biophysics, Washington University School of Medicine, St. Louis, MO USA

**Keywords:** Medical student, Nutrition, Lifestyle choices, Education

## Abstract

**Background:**

Poor diet and lifestyle are associated with elevated risk of poor health outcomes. Medical students (MS) learn about health promotion counseling and the importance of dietary, exercise, and sleep habits. Research suggests that such counseling is more impactful for patients when providers themselves model healthy choices. There is little evidence evaluating whether MS themselves adopt the lifestyles they teach their patients.

**Methods:**

We utilized a cross-sectional approach to conduct a voluntary, anonymous survey of all enrolled students at one allopathic medical school in the northeast. Descriptive, bi-and multivariate logistic regression analyses were used to characterize MS lifestyle habits across four class cohorts.

**Results:**

Overall response rate (RR) was 46% (401/874). MS did not eat as healthy (70%) nor exercise as much as they would like (79%) and perceived their diet (48%) and exercise (57%) habits as healthier before medical school. MS in class years 2–4 vs. class year 1 were nearly 2.5 × more likely to consume vegetables daily (p < 0.01). Barriers included time constraints (88%), cost (62%), and fatigue (73%).

**Conclusion:**

This is the first study to survey first through fourth-year MS regarding their dietary, exercise, and sleep habits. We identified a significant decline in MS self-reported healthy lifestyle choices and subjective levels of health satisfaction compared to prematriculation statuses. Most respondents cited time constraints, fatigue, and financial limitations as barriers. Future research should further investigate reported barriers and develop approaches to their mitigation. Results must be interpreted in the context of survey limitations, including response rate, selection, and recall biases.

**Supplementary Information:**

The online version contains supplementary material available at 10.1007/s40670-026-02718-3.

## Background

Poor diet and sedentary lifestyles are significant contributors to obesity, cardiovascular disease, type 2 diabetes mellitus and other leading causes of morbidity and mortality [1, 2]. In contrast, adequate exercise, sufficient sleep [3], and diets rich in minimally processed plant-based foods, fish, unsaturated fats, nuts, and legumes have been shown to reduce rates of coronary artery disease, ischemic stroke, and total cardiovascular disease [4]. Given the immense impact of such lifestyle choices on health outcomes, medical professionals are expected to counsel patients on these factors. This expectation also carries broader public health significance, as physician-led prevention efforts contribute to improved population health and reduced disease burden.

For most physicians as well as resident/fellow trainees, the foundation for lifestyle counseling is developed in medical school. While nutritional education in United States medical schools generally remains rudimentary [5], Reports suggest that United States medical schools' nutrition-related curricula typically includes basic nutritional science and ‘heart-healthy’ lifestyle guidelines [6, 7] reinforced by integrated biochemistry, cardiovascular, and pharmacology courses [8] and ultimately assessed on the United States Medical Licensing Exams (Steps 1 & 2) [9]. Despite this, there is little known about whether Medical Students (MS) in the United States adopt the lifestyle and behaviors they are trained to promote [10] as a Social Modeling Theory would support individuals as more likely to adopt behaviors demonstrated by credible role models [11]. Similarly, the Self-Determination Theory emphasizes that autonomy, competence, and interpersonal connection support sustained behavior change. If MS do not internalize and model recommended behaviors themselves, their effectiveness in motivating patients may be diminished.

Understanding whether MS model recommended healthy lifestyle practices is essential particularly given the Liaison Committee on Medical Education’s interest in strengthening nutrition curriculum in medical schools [12]. Literature demonstrates that physicians who adhere to lifestyle recommendations are more effective and confident in counseling patients on these matters [13–16]. Further research shows that low-threshold (short lecture series) efforts to positively influence MS’ dietary and lifestyle choices [17]. It is therefore critical to examine physician lifestyle habits and behaviors, as they may impact both the validity and strength of their patient interactions—ultimately contributing to health outcomes.

Although undergraduate medical education can increase MS competency in delivering such counseling [18], available reports evaluating MS personal lifestyle practices are limited. MS in the U.S. are reported to typically have better health and fitness than their same-aged peers [19], however, they often fail to maintain healthy dietary choices [20–22]. One study found that by the end of their first year, MS experienced declines in physical activity, sleep, general health, and increased difficulty in maintaining a balanced diet when compared to responses prior to matriculation [23]. No studies have systematically compared lifestyle habits across all four years of medical school or investigated specific barriers that influence these choices.

Our study aimed to fill these gaps by simultaneously assessing dietary, exercise, and sleep trends across first- through fourth-year medical students (MS1, MS2, MS3, MS4) at an allopathic medical school in the northeastern United States. This student-led initiative aimed to examine perceived barriers experienced by MS and compare self-reported satisfaction with their physical/emotional health, stress levels, wellness, sleep, and dietary habits before and during medical school. Lastly, we propose strategies to mitigate these barriers—with the broader goal of enhancing both physician well-being and patient care.

## Methods

### Study Design

A cross-sectional research design was used to survey enrolled MS at New York Medical College (NYMC), an allopathic medical school in Valhalla, New York (Supplement 1). All MS1, MS2, MS3, and MS4 students were invited to participate via email, GroupMe chat, and scannable QR codes presented during class discussions from March to May 2024. Participation in the survey was voluntary, and results were de-identified to maintain anonymity. Three gift cards (two $50 and one $100) were awarded via random drawing to incentivize participation.

The survey tool was piloted in a sample of MS3 students (n = 15) to determine feasibility of data collection, ease of use, and anonymity prior to survey distribution electronically using Qualtrics Research Suite (Qualtrics^XM^ Seattle, WA). Pilot data were excluded from the survey results, and no authors of the study participated in the survey.

The survey assessed student demographic data (class year, gender- and racial-identity); the frequency, duration, and type of exercise in which students engage; whether students felt their habits had improved, remained unchanged, or worsened since matriculating in medical school; and barriers experienced when trying to make healthy lifestyle choices. Additional survey questions were adopted from the Mayo Clinic and American Heart Association Mini-EAT 9-item Rapid Dietary Screener—a validated dietary screening measure correlated with cardiovascular health predictions [24]. Lastly, using a scale of 1–10, students were asked to rank their perceptions of physical and emotional health, stress, and social support during medical school as compared to prior to enrollment, where 10 was defined as extremely satisfied and 1 was defined as extremely dissatisfied.

This study was granted an exemption by the NYMC Institutional Review Board and endorsed by NYMC administration.

### Data Analysis

Data were extracted from Qualtrics and imported to Stata SE 18.5 for statistical analysis. The main exposure was MS class year (MS1, MS2, MS3 and MS4). The main outcomes were (1) dietary habits (consumption of fruit, vegetable, legumes and nuts, seafood, whole grains, refined grains, low-fat dairy, high-fat dairy, and sweets), coded as 0 = "I do not eat it at all to consumption of 2 servings per week," 1 = "consumption of 3 to 6 servings per week," and 2 = "consumption of 1 or more servings per day”; (2) exercise hours per week, coded as 0 = "0–1 h: Below Recommended" and 1 = "2 or more hours: Recommended [25]”; and (3) sleep hours, coded as 0 = "2–6 h: Inadequate Sleep" and 1 = "7 or more hours: Adequate Sleep [26].” Dietary variables were recorded as binary outcomes (0 = “ < 7 servings per week: not daily” and 1 = “1 or more serving per day: daily”) for regression analysis.

Descriptive statistics were calculated to assess dietary habits and barriers to a healthy diet, physical activity levels and barriers to exercise, sleep habits, and the student’s overall satisfaction level with their physical and mental wellbeing both prior to medical school and at the time they filled out the survey. Descriptive statistics were presented as frequencies and percentages. The Wilcoxon signed-rank test and additional paired t-tests were used to examine survey question responses addressing health and wellbeing satisfaction before and during medical school.

Logistic regression was utilized to assess the association between the exposure variable (class year) and outcomes (dietary habits, exercise hours, and sleep hours). Unadjusted and adjusted odds ratios (OR) with corresponding 95% confidence intervals and p-values were reported. Following the logistic regression analysis for the outcome of daily vegetable consumption, predictive probabilities were calculated to estimate the probability of daily vegetable consumption at different levels of main exposure (MS1, MS2, MS3, and MS4). These predictive probabilities were then compared to assess the statistical significance of differences between groups, with a particular focus on comparing MS2, MS3 and MS4 with MS1, using an alpha level adjusted to account for multiple comparisons. All statistical tests were two-sided, with significance set at an alpha level of 0.05, applying Bonferroni corrections for multiple comparisons when appropriate.

## Results

Of the 874 MS who received the survey, 401 (46%) voluntarily completed the survey after excluding 146 entries characterized as ‘testing trial’ and unfinished surveys. Of the 401 students who completed the survey, 157 (39%) identified as male, and 236 (59%) identified as female. The participants’ distribution across class cohorts was as follows: 111 (28%) MS1, 108 (27%) MS2, 98 (24%) MS3, and 84 (21%) MS4 (Table 1).

**Table 1:** Descriptive characteristics of the study population at NYMC (n = 401)

Total Study Population	401	
Demographics	n	%
Sex		
Male	157	39.15
Female	236	58.85
Other/missing	8	2.00
Race		
White	197	49.13
Asian	121	30.17
Black	22	5.49
Hispanic	16	3.99
Other/biracial	40	9.98
Missing	5	1.25
Medical Student Class		
MS1 (Class of 2027)	111	27.68
MS2 (Class of 2026)	108	26.93
MS3 (Class of 2025)	98	24.44
MS4 (Class of 2024)	84	20.95

The study population included students across all four class years with representation across gender and racial groups

### Dietary Trends

**Fig. 1:**
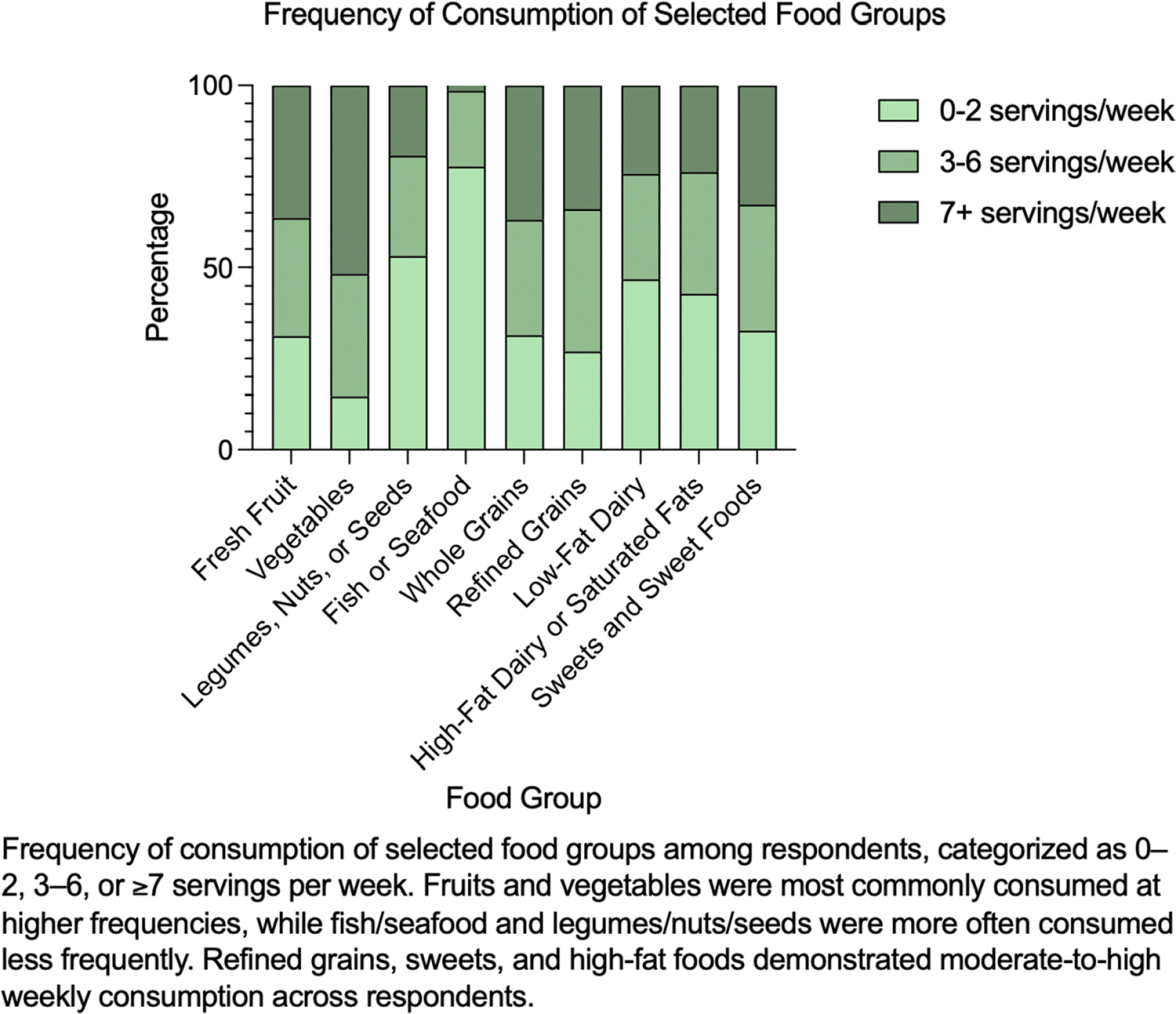
Frequency of Consumption of Selected Food Groups

Diet was assessed using the validated Mini-EAT 9-item Rapid Dietary Screener both across the entire sample population and within each class subgroup. Overall, most students reported consuming less than one serving per day of fresh fruit (64%), legumes/nuts/seeds (80%), whole grains (64%), and low-fat dairy (76%). However, more than half of the respondents consumed more than one serving per day of vegetables (52%). Most students also reported consuming less than one serving per day of traditionally lower-quality foods including refined grains (66%), high-fat dairy and saturated fat (76%), and sweets (67%)—with this trend varying by class year (Table 2, Fig. 1).

**Table 2:** Additional descriptive characteristics of lifestyle behaviors among the study population at NYMC (n = 401)

Total Study Population	401	
Eating habit	n	%
How often do you eat fresh fruit		
I do not eat it at all-2 servings per week	125	31.17
3–6 servings per week	130	32.42
1 + serving per week	146	36.41
How often do you eat vegetables		
I do not eat it at all-2 servings per week	59	14.71
3–6 servings per week	135	33.67
1 + serving per week	207	51.62
How often do you eat legumes, nuts, or seeds		
I do not eat it at all-2 servings per week	213	53.12
3–6 servings per week	111	27.68
1 + serving per week	77	19.2
How often do you eat fish or seafood		
I do not eat it at all-2 servings per week	312	77.81
3–6 servings per week	83	20.7
1 + serving per week	6	19.2
How often do you eat whole grains		
I do not eat it at all-2 servings per week	126	31.42
3–6 servings per week	127	31.67
1 + serving per week	148	36.91
How often do you eat refined grains		
I do not eat it at all-2 servings per week	108	26.93
3–6 servings per week	157	39.15
1 + serving per week	136	33.92
How often do you eat low-fat dairy		
I do not eat it at all-2 servings per week	188	46.88
3–6 servings per week	116	28.93
1 + serving per week	97	24.19
How often do you eat high-fat dairy and saturated fat		
I do not eat it at all-2 servings per week	172	42.89
3–6 servings per week	134	33.42
1 + serving per week	95	23.69
How often do you eat sweets and sweet foods		
I do not eat it at all-2 servings per week	131	32.67
3–6 servings per week	139	34.66
1 + serving per week	131	32.67

This table summarizes baseline patterns related to diet, highlighting variability in health-related behaviors among the surveyed population

When data were examined within subgroups, consumption of vegetables was significantly higher for MS2, MS3, and MS4 compared to MS1 (p < 0.01). Fruit and whole grain consumption also trended higher among upperclassmen than MS1 (p = ns). Upperclassmen demonstrated a trend toward consuming more high-fat dairy and sweets compared to MS1 (p = ns), while refined grain consumption trended less among MS3 and MS4 compared to MS1 (p = ns). These trends remained after controlling for race and gender identity (Table 3).

**Table 3:** Unadjusted and adjusted odds ratios (ORs) with 95% confidence intervals for lifestyle outcomes by medical school class year

		Unadjusted OR (95% CI)	p	Adjusted OR (95% CI)	p
Fruits					
	MS2 vs MS1	1.56 (0.89, 2.72)	0.119	1.53 (0.86, 2.73)	0.148
	MS3 vs MS1	1.20 (0.67, 2.15)	0.532	1.20 (0.66, 2.18)	0.554
	MS4 vs MS1	1.54 (0.85, 2.79)	0.154	1.74 (0.93, 3.28)	0.084
Vegetables					
	MS2 vs MS1	2.30 (1.34, 3.95)	** < 0.01**	2.23 (1.26, 3.94)	** < 0.01**
	MS3 vs MS1	2.19 (1.26, 3.81)	** < 0.01**	2.44 (1.36, 4.40)	** < 0.01**
	MS4 vs MS1	1.99 (1.12, 3.54)	** < 0.05**	2.44 (1.30, 4.58)	** < 0.01**
Legumes/Nuts					
	MS2 vs MS1	0.70 (0.35, 1.43)	0.330	0.60 (0.29, 1.27)	0.181
	MS3 vs MS1	1.31 (0.78, 2.43)	0.417	1.24 (0.62, 2.47)	0.538
	MS4 vs MS1	0.88 (0.42, 1.82)	0.729	0.97 (0.44, 2.10)	0.929
Wholegrains					
	MS2 vs MS1	1.13 (0.64, 1.99)	0.668	1.03 (0.57, 1.87)	0.912
	MS3 vs MS1	1.38 (0.78, 2.43)	0.274	1.31 (0.72, 2.38)	0.376
	MS4 vs MS1	1.79 (1.00. 3.23)	0.051	1.98 (1.05, 3.72)	** < 0.05**
Refined Grains					
	MS2 vs MS1	1.43 (0.82, 2.48)	0.203	1.43 (0.81, 2.55)	0.219
	MS3 vs MS1	0.88 (0.49, 1.58)	0.674	0.96 (0.52, 1.76)	0.888
	MS4 vs MS1	0.80 (0.43, 1.48)	0.478	0.86 (0.44, 1.65)	0.645
Low-fat Dairy					
	MS2 vs MS1	1.89 (1.01, 3.53)	** < 0.05**	1.79 (0.93, 3.44)	0.079
	MS3 vs MS1	0.96 (0.48, 1.94)	0.919	0.97 (0.47, 1.99)	0.933
	MS4 vs MS1	1.82 (0.93, 3.54)	0.079	1.74 (0.86, 3.51)	0.121
High-fat Dairy					
	MS2 vs MS1	1.70 (0.91, 3.18)	0.094	1.79 (0.93, 3.43)	0.079
	MS3 vs MS1	1.31 (0.68, 2.53)	0.417	1.29 (0.65, 2.55)	0.462
	MS4 vs MS1	1.03 (0.51, 2.08)	0.942	1.09 (0.52, 2.29)	0.821
Sweets					
	MS2 vs MS1	1.23 (0.70, 2.19)	0.472	1.22 (0.67, 2.21)	0.517
	MS3 vs MS1	1.43 (0.80, 2.56)	0.224	1.43 (0.65, 2.62)	0.248
	MS4 vs MS1	1.17 (0.63, 2.16)	0.618	1.66 (0.61,221)	0.655
Exercise					
	MS2 vs MS1	0.64 (0.35, 1.14)	0.131	0.59 (0.32, 1.08)	0.090
	MS3 vs MS1	0.63 (0.35, 1.15)	0.134	0.66 (0.35, 1.21)	0.178
	MS4 vs MS1	1.86 (0.90, 3.87)	0.095	1.69 (0.81, 3.55)	0.163
Sleep					
	MS2 vs MS1	0.97(0.56, 1.67)	0.911	0.90 (0.51, 1.59)	0.719
	MS3 vs MS1	0.57 (0.32, 1.00)	** < 0.05**	0.55 (0.31, 1.00)	** < 0.05**
	MS4 vs MS1	1.23 (0.67, 2.24)	0.501	1.21 (0.65, 2.25)	0.549

Adjusted models controlled for race and gender for all analyses in addition to potential barriers for dietary outcomes. Consumption of vegetables was higher for MS2, MS3, and MS4 compared to MS1. MS3 reported significantly less sleep compared to MS1 participants

**Fig. 2:**
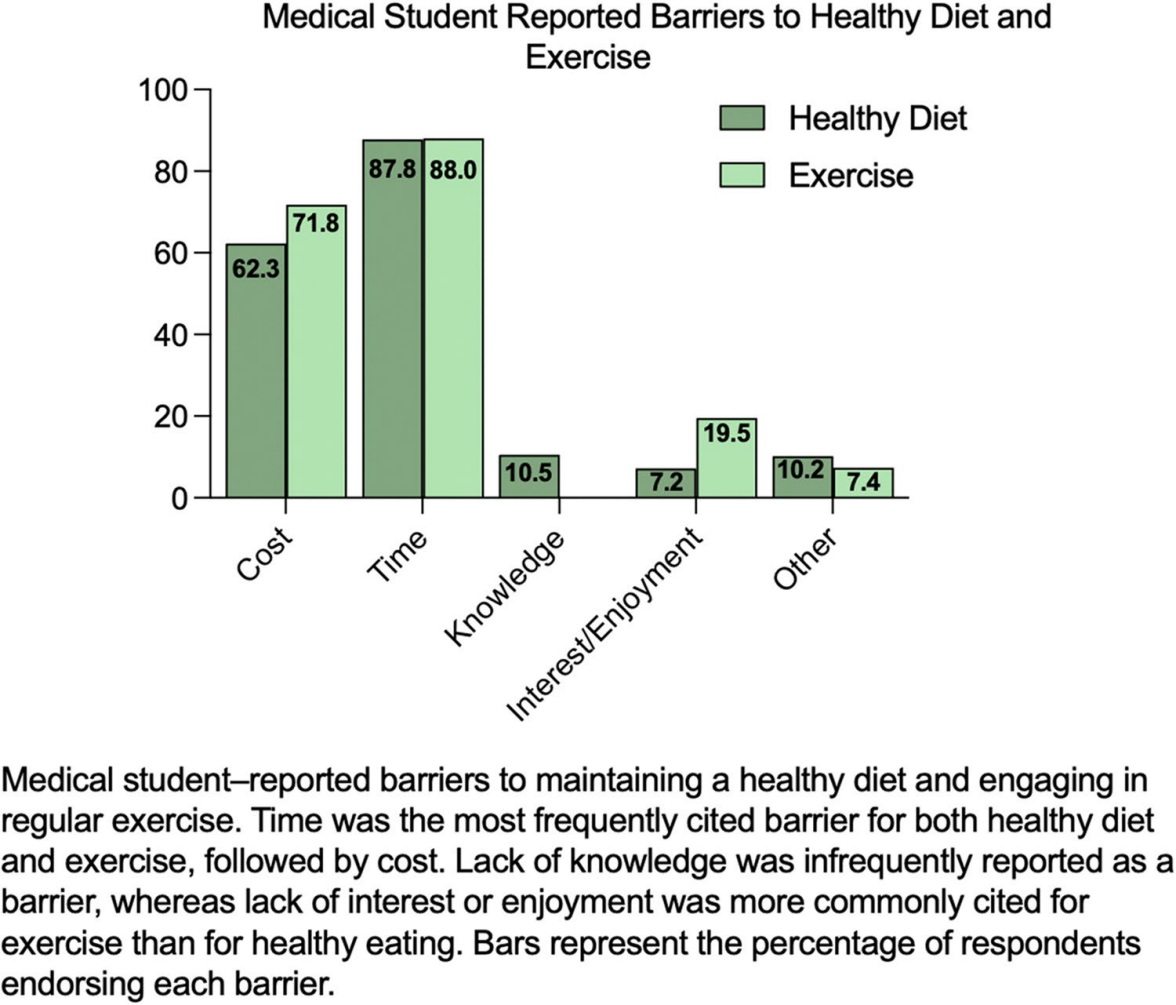
Medical student reported barriers to healthy diet and exercise

The most reported overall barriers to healthy eating were lack of time (88%) and cost (62%). Lack of knowledge about how to cook “healthy” food (10%) and limited interest in cooking “healthier” (7%) were also notable barriers. Most students (65%) reported experiencing more than one barrier to healthy eating (Table 4, Fig. 2).

**Table 4:** Eating habits, perceived barriers to healthy eating, and self-reported changes in dietary behaviors before and during medical school

Total Study Population		401	
Eating habit		n	%
Do you eat as ‘healthy’ as you would like to			
No		280	69.83
Yes		121	30.17
Eat prior vs. in medical school			
diet was healthier than now		192	47.88
My diet is unchanged		114	28.43
Healthier diet while in medical school		95	23.69
Barriers to eating healty			
Cost		16	3.99
Lack of time		102	25.44
Lack of knowledge		2	0.5
No interest		6	1.5
Other barriers		13	3.24
More than one barriers		262	65.34
Barrier-Lack of time to cook			
No		359	89.53
Yes		42	10.47
Barrier-No interest in cooking ‘healthier’			
No	No	372	92.77
Yes	Yes	29	7.23
Barrier-Other barriers not listed			
No	No	360	89.78
Yes	Yes	41	10.22

Students commonly reported time constraints and cost as barriers to healthy eating. A majority perceived their eating habits to worsen during medical school compared to before matriculation, underscoring the impact of training-related demands on dietary behaviors

A strong majority of students (87%) cited a medium or high satisfaction with the quality of their nutrition education. However, only 24% of all respondents reported implementing a subjectively healthier diet in medical school when compared to their diet before matriculating (Table 4). Seventy percent of MS reported that their diet is not as “healthy” as they would like it to be, and results demonstrated a significant decrease in student satisfaction compared to their diet before matriculating (p < 0.0001) (Table 5).

**Table 5:** Medical student perception of physical, emotional, combined physical/emotional health, overall wellness, diet satisfaction, and stress levels before and during medical school

Variables	z-value	p-value	Conclusion
Physical Health satisfaction	−6.978	< 0.0001	Decreased
Emotional Health Satisfaction	−3.12	0.0018	Decreased
Combined Physical & Emotional Health Satisfaction	−5.613	< 0.0001	Decreased
Overall Wellness Satisfaction	−8.395	< 0.0001	Decreased
Diet Satisfaction	−6.423	< 0.0001	Decreased
Stress Levels	9.05	< 0.0001	Decreased

Wilcoxon signed-rank tests demonstrated significant declines in physical health satisfaction, emotional health satisfaction, combined physical/emotional health, overall wellness, and diet satisfaction during medical school compared to before matriculation (all p < 0.01). In contrast, perceived stress levels increased significantly during medical school, highlighting a marked deterioration in multiple dimensions of student well-being over the course of training

### Exercise Trends

Twenty-eight percent of respondents reported engaging in one hour or less of exercise per week and less than half (41%) reported exercising 4 or more hours per week. Most (59%) MS did not meet the exercise recommendations set forth by the American Heart Association (150 min or more per week) [25].Walking/running (72%) was cited as the most common form of exercise followed by weight training (52%), resistance training (33%), Yoga/Pilates (27%), organized team sports (14%), and other (10%). Seventy percent of respondents engaged in more than one type of exercise. Most students (53%) reported exercising three or more days out of the week preceding the survey and 16% reported that they did not exercise at all in the preceding week.

When each respective class was compared in aggregate to the other cohorts, MS2 and MS3 students tended to exercise less than MS1 (p = ns) while MS4 tended to exercise the most of all class years (p = ns) (Table 4).

Significant barriers to exercise included time (88%) and fatigue (73%). Cost (28%), lack of enjoyment (19%), lack of organized sports (17%), and other (7%) were also reported, with 79% of students reporting more than one barrier (Fig. 2). In addition, 79% of students reported that they do not exercise as much as they desire. Only 20% reported an increase in their exercise habits in medical school compared to their habits before matriculating. Respondents reported a significant decrease in overall satisfaction with their personal level of physical health compared to their health before matriculating (p < 0.0001) (Table 3).

### Sleep and Wellness Trends

Overall, 50% of students reported getting less sleep in medical school than before matriculating and nearly half of respondents reported feeling poorly rested the majority of days (4 or more) per week. When they were asked to compare their respective health prior to beginning medical school, students reported significantly decreased satisfaction in emotional health and wellness during medical school (p < 0.01).

When comparing sleep patterns across class cohorts we found that MS3 were less likely to get adequate sleep (7 or more hours) compared to MS1 (p < 0.01), and MS4 reported the most amount of sleep (p = ns).

## Discussion

This study is the first of its kind to use a validated dietary assessment tool to examine the intersection of diet, sleep, and exercise among MS across all four years of education at an allopathic medical school in the United States. Our findings highlight a persistent challenge in undergraduate medical education, that is, while students receive instruction on healthy lifestyle practices, and some might practice counseling patients (both standardized and during clinical rotations) on these practices, students struggle to implement these behaviors in their own lives.

We found that medical student dietary habits were consistently suboptimal across all four class years. Specifically, students reported consuming less than one serving per day of fresh fruits, legumes, nuts, seeds, whole grains, and low-fat dairy. Although upperclassmen reported increased vegetable intake and decreased consumption of refined grains, they also showed increased consumption of high-fat dairy and sweets, reflecting mixed trends rather than a clear trajectory of dietary improvement over time. These mixed trends may be explained by a host of factors, including but not limited to time constraints experienced in third year clerkships and stressors of applying to residency in the fourth year. Moreover, only one in four students reported adopting a healthier diet during medical school, and the majority expressed dissatisfaction with their current eating habits.

Exercise patterns were also consistently suboptimal as most students did not meet the American Heart Association’s recommended exercise guidelines [25]. However, the exercise trends should be interpreted in the context of the time constraints that MS consistently face. Sleep behaviors also reflected strain, with half of respondents reporting less sleep than before medical school, and nearly half feeling poorly rested on four or more days per week.

These findings are concerning given the well-documented benefits of maintaining healthy lifestyle behaviors during medical training. Studies show that MS well-being is positively associated with academic achievement and self-reported empathy [27, 28]. Moreover, physicians who model healthy habits are more likely to counsel patients effectively on these behaviors. Thus, failing to support MS in prioritizing their own health may ultimately undermine both their personal well-being and professional effectiveness [29]. These patterns often persist and intensify during residency and beyond, highlighting the importance of early and sustained intervention [30, 31].

To support students more effectively, medical schools may consider actively addressing the barriers impeding healthy behaviors. To bridge the gap between knowledge and practical application, medical curricula should integrate proactive, skills-based training—particularly in nutrition. This could include instruction on meal planning, budgeting for healthy eating, understanding the health impacts of different dietary patterns, and effective strategies for patient counseling [32, 33]. Partnering with dietetics programs could also provide invaluable interprofessional learning opportunities, enhancing both student group's education and their ability to collaborate. Medical schools may consider investing in culinary medicine programs that teach students cooking methodologies centered around the Mediterranean diet [34, 35]. Such programs could also educate students about the importance of understanding the Nutrition Facts food labels of a product they wish to consume and help them adapt to certain dietary restrictions. Further, the AAMC might consider recommending a nationwide curriculum, perhaps centered on medical student dietary and lifestyle choices, to ensure all students receive basic guidance on how to care for themselves as they learn to care for others. In addition, gamification efforts have proven successful in other areas of nutrition education and could be explored in medical education [36, 37].

Exercise promotion could be similarly prioritized. Facilitating access to subsidized gym memberships, group fitness classes, and intramural sports would encourage physical activity. Incorporating creative, time-efficient approaches—such as encouraging students to watch asynchronous lectures while on a treadmill or stationary bike—could further lower participation barriers. Institutions might also consider implementing physical education requirements, as is common in many undergraduate programs, or organizing friendly competitions to foster a culture of wellness and accountability among students for making healthy choices.

Time constraints remain the most reported and most difficult barrier to developing healthy lifestyle habits; alleviating this will require systemic solutions. Given the large volume of content MS must master in a short period of time in medical school, this is not entirely unexpected. Freeing up hours in a medical student's day might not be the most realistic solution. However, during the pre-clerkship phase, improving lecture efficiency through flipped-classroom models or targeted asynchronous content may help students reclaim valuable time. During clinical years, reducing commute times—such as through institutionally supported housing near hospital sites—could further alleviate time-related stressors. These measures might create more opportunities for healthy eating, physical activity, and increase time for sleep. Throughout medical school, time management is often introduced in the context of efficient and effective study methods. However, this skill should be taught and interpreted in the context of the whole person when possible by directly addressing diet, sleep, and exercise within these sessions.

Wellness education should be introduced early and reinforced consistently throughout medical school, allowing students to internalize and apply health-promoting practices as they progress. Addressing logistical barriers during medical school—such as time constraints, cost, and fatigue—will be crucial as these challenges were most reported during the MS2 and MS3 years, when academic and clinical demands peak. Interventions could target time management, streamlined access to wellness resources, and structural support for self-care to equip students to maintain healthier habits during the most demanding phases of their training.

This study has several limitations. As a single-institution, cross-sectional study, the findings may not be generalizable, and the design precludes direct comparison between class years and an inability to establish causality. Selection bias may have affected participation, with students particularly interested in health more likely to complete the survey. Recall bias may have influenced responses about pre-matriculation behaviors, given that the pre-medical school matriculation period was up to four years prior for some respondents (MS4).

Future research should expand to multiple institutions to assess whether these trends persist across diverse curricula and learning environments. Longitudinal studies following the same cohort over time would clarify how lifestyle habits evolve throughout medical school. Broadening the scope to include residents, fellows, and attending physicians would provide valuable insight into how these behaviors change throughout a medical career. Moreover, future studies may examine in greater detail the correlation between medical trainees’ lifestyle habits and their efficacy in counseling patients. Finally, further exploration of the relationships between diet, physical activity, sleep, stress, and self-reported physical and emotional health with objective measurements and deeper exploration of barriers may offer a deeper understanding and inform more targeted interventions.

## Conclusion

While medical knowledge and healthy lifestyle counseling skills improve throughout the duration of medical school, our findings show that students often fail to adopt the behaviors they are trained to promote, which contributes to declining health satisfaction during medical school. Medical students face multiple barriers—including time, financial resources, and fatigue—that seem to impede their ability to prioritize dietary choices, exercise, and sleep. This disconnect has implications for the health outcomes of future physicians and the quality and efficacy of lifestyle counseling delivered to patients. These findings highlight actionable opportunities for further investigation and targeted medical education interventions such as structured wellness curricula, accessible nutrition and fitness resources, and systems-level efforts to free up valuable time. Further research should investigate which strategies most effectively mitigate these barriers, with the goal of fostering an educational environment that supports personal health, enhances professional preparedness, and strengthens patient care. Ultimately, strengthening physician well-being carries broader public health benefits as healthier clinicians are better equipped to model preventive behaviors, advocate for community wellness, and contribute to a more resilient healthcare workforce.

## Supplementary Information

Below is the link to the electronic supplementary material.

Supplementary file1 (DOCX 24 KB)

## Abbreviations

MS: Medical Student
RR: Response Rate
MS1-4: First-Fourth Year Medical Student
NYMC: New York Medical College
OR: Odds Ratio
