# Supplementary material for: Do They Practice What They Preach? A Cross-Sectional Study of Heart-Healthy Dietary, Exercise, and Sleep Habits in US Medical Students
Source: Med Sci Educ. 2026 Apr 11;36(3):1295–304. doi: 10.1007/s40670-026-02718-3 (PMC13356121; doi:10.1007/s40670-026-02718-3)
Supplement: Supplementary file 1 — Supplementary file1 (DOCX 24 KB) [file 40670_2026_2718_MOESM1_ESM.docx]

**Do They Practice What They Preach? A Cross-Sectional Study of Heart-Healthy Dietary, Exercise, and Sleep Habits in US Medical Students**

**Journal: Medical Science Educator**

**Authors**: Harris Ziggy Whiteson ^1^, Madison Drogy^1^, David Haner Wasserstein^1^, Jane Qingyi Chen^1^, Jay Ayar BDS MPH ^2^, Elizabeth Drugge PhD MPH ^2^, Kathryn Spanknebel MD^3^, William H. Frishman MD^4^, Kristina H. Petersen PhD^5^

**Affiliations**:

^1^New York Medical College, School of Medicine, Valhalla, NY

^2^Department of Public Health, School of Health Sciences and Practice, New York Medical College, Valhalla, New York

^3^Department of Surgery, New York Medical College, School of Medicine, Valhalla, NY

^4^Department of Medicine, New York Medical College, School of Medicine, Valhalla, NY ^5^Department of Biochemistry & Molecular Biophysics, Washington University School of Medicine, St. Louis, MO USA

**Corresponding Author**

Harris Z. Whiteson

646-599-2136

hwhiteso@student.nymc.edu

**Survey Questionnaire: A Cross-Sectional Study of Dietary Habits, Nutritional Status, and Lifestyle Choices in Medical Students**

Description: You are invited to participate in a research study on the dietary habits, nutritional status, and lifestyle choices in medical students at New York Medical College, School of Medicine (NYMC SOM). The purpose of this research is to evaluate dietary habits, physical activity levels, and overall lifestyle choices of United States Medical Students, who, despite their extensive knowledge of healthy practices, often encounter challenges in implementing them. You will be asked to complete an anonymous survey answering questions about your experiences in medical school related to living a ‘heart healthy’ lifestyle. You will only be asked to complete this survey once. You have been invited to participate in this research as you are a medical student at NYMC SOM. We expect to enroll approximately 200 participants in this study. Your participation in this study is voluntary. Your answers will remain completely anonymous. It will not be possible to trace the answers back to you in any way.

Time involvement: Your participation will take approximately 10 minutes to complete this survey.

Are there benefits to taking part in the study? You are not expected to directly benefit from your participation in the research study. However, we hope the information learned from this study will contribute to our understanding of barriers that medical students might face when trying to lead a healthy lifestyle.

Are there risks to taking part in this study? The risks associated with the study are being uncomfortable with some of the questions posed.

Who is conducting this study? This study is being conducted by researchers at New York Medical College.

Will you be paid for participating? You will not be paid for participating.

What are my rights as a research participant? Your participation in this study is voluntary. You may decline to respond to any questions in the survey that you do not wish to answer for any reason. The results from your survey will remain anonymous. Identifiable private information is not collected. The information from your survey could be used for future studies or distributed to another investigator for future research studies without additional informed consent.

For questions about the study Questions: If you have any questions, concerns or complaints about this research study, its procedures, risks and benefits, you should contact the Study Coordinator, Harris Whiteson at 646-599-2136

- I have read this consent form and agree to be in this study, with the understanding that I may withdraw at any time. By continuing on with the survey I have given my consent to participate.

What is your racial identity?

What is your gender identity?

What is your age?

What class are you?

- MS1 (Class of 2027)
- MS2 (Class of 2026)
- MS3 (Class of 2025)
- MS4 (Class of 2024)

**Section 1: Dietary Habits (Questions adopted from American Heart Association Mini-EAT 9 Assessment Tool**

1. How often do you eat fresh fruit? (One serving is equal to one small apple, one half of a banana, one cup of mandarin oranges, one cup of melon, one cup of raspberries, three quarters of a cup of blueberries, one and a half cup of strawberries)

- I do not eat it at all
- Less than one serving per week
- 1-2 servings per week
- 3-4 servings per week
- 5-6 servings per week
- 1 serving per day
- 2-3 servings per day
- 4-5 servings per day
- 6 or more servings per day

1. How often do you eat vegetables? (One serving is equal to one cup of raw vegetables or or one half cup of cooked vegetables)

- I do not eat it at all
- Less than one serving per week
- 1-2 servings per week
- 3-4 servings per week
- 5-6 servings per week
- 1 serving per day
- 2-3 servings per day
- 4-5 servings per day
- 6 or more servings per day

1. How often do you eat legumes, nuts, or seeds? (One serving is equal to one half cup of cooked legumes or seeds, one third cup of hummus or bean dip, one half cup of tofu, one fourth cup of tempeh, a handful of seeds)

- I do not eat it at all
- Less than one serving per week
- 1-2 servings per week
- 3-4 servings per week
- 5-6 servings per week
- 1 serving per day
- 2-3 servings per day
- 4-5 servings per day
- 6 or more servings per day

1. How often do you eat fish or seafood? (One serving is equal to three ounces of cooked or canned fish, a palm sized serving of raw fish)

- I do not eat it at all
- Less than one serving per week
- 1-2 servings per week
- 3-4 servings per week
- 5-6 servings per week
- 1 serving per day
- 2-3 servings per day
- 4-5 servings per day
- 6 or more servings per day

1. How often do you eat whole grains? (One serving is equal to one slice of whole grain bread, one half cup of cooked cereal, one half cup of cooked brown rice or whole grain pasta, one small corn tortilla, one half cup of cooked grits, one cup of ready-to-eat cereal flakes)

- I do not eat it at all
- Less than one serving per week
- 1-2 servings per week
- 3-4 servings per week
- 5-6 servings per week
- 1 serving per day
- 2-3 servings per day
- 4-5 servings per day
- 6 or more servings per day

1. How often do you eat refined grains? (One serving is equal to one slice of white bread, one half of a roll, one half of a small white bagel of english muffin, one half cup of cooked white rice or pasta, one small wheat tortilla)

- I do not eat it at all
- Less than one serving per week
- 1-2 servings per week
- 3-4 servings per week
- 5-6 servings per week
- 1 serving per day
- 2-3 servings per day
- 4-5 servings per day
- 6 or more servings per day

1. How often do you eat low-fat dairy? (One serving is equal to one cup of low fat or skim milk, three quarters of a cup of low fat yogurt, one pre-packaged slice of low-fat cheese, one and one half ounce of mozzarella)

- I do not eat it at all
- Less than one serving per week
- 1-2 servings per week
- 3-4 servings per week
- 5-6 servings per week
- 1 serving per day
- 2-3 servings per day
- 4-5 servings per day
- 6 or more servings per day

1. How often do you eat high-fat dairy and saturated fat? (One serving is equal to one cup of 2% or whole milk, three quarters of a cup of yogurt, one pre-packaged slice of cheese, two ounces of processed cheese, one half cup of ice cream, one teaspoon of butter, shortening, or coconut oil)

- I do not eat it at all
- Less than one serving per week
- 1-2 servings per week
- 3-4 servings per week
- 5-6 servings per week
- 1 serving per day
- 2-3 servings per day
- 4-5 servings per day
- 6 or more servings per day

1. How often do you eat sweets and sweet foods? (One serving is equal to one and a half ounces of gummy candy, three pieces of hard candy, one small piece of cake or pastry, one medium doughnut or sweet snack, two to three sweet biscuits or cookies)

- I do not eat it at all
- Less than one serving per week
- 1-2 servings per week
- 3-4 servings per week
- 5-6 servings per week
- 1 serving per day
- 2-3 servings per day
- 4-5 servings per day
- 6 or more servings per day

1. Do you eat as ‘healthy’ as you would like to?

- Yes
- No

1. Do you feel as though you ate ‘healthier’ in medical school or did you eat ‘healthier’ prior to attending?

- I eat a ‘healthier’ diet while in medical school
- Prior to attending medical school, my diet was ‘healthier’ than it is now

1. What are current barriers to eating healthy that you experience as a medical student? (you may select more than one answer)

- Cost
- Lack of time to cook
- Lack of knowledge about how to cook ‘healthy’ food
- No interest in cooking ‘healthier’
- Other

**Section 2: Exercise**

1. Approximately how many hours per week do you exercise?

- 0-1
- 2-3
- 4-5
- 6-7
- 8+

1. What is your preferred exercise training?

- Weight training (bench press, dumbbells, etc)
- Resistance training (body weight workouts, dips, pullups, chinups, etc)
- Walking/running
- Organized team sport
- Yoga/Pilates
- Other

1. During the last seven days, on how many days did you participate in moderate physical activities for at least ten minutes? (Moderate refers to physical activities that make you breathe somewhat harder than normal and can make you feel fatigued)

- 0
- 1
- 2
- 3
- 4
- 5
- 6
- 7

1. Do you exercise as much as you would like to?

- Yes
- No

1. Do you exercise more or less in medical school than you did prior to attending?

- More
- Same Amount
- Less

1. What are current barriers to exercise that you experience as a medical student? (you may select more than one answer)

- Price to access facilities
- Lack of enjoyment in exercising
- Fatigue from other responsibilities (studying, cooking, etc…)Lack
- Time constraints
- Lack of organized sports/exercise classes
- Other

**Section 3: Habits - this section contains sensitive questions. This survey is 100% anonymous and there are no ways for us to track answers back to you.**

1. On an average week, approximately how much time do you spend socializing with friends?

- 0-1 hours
- 2-3 hours
- 4-5 hours
- 6-7 hours
- 8 hours plus

1. During an average month, how many times do you see friends who are not in medical school?

- 0-1 times
- 2-3 times
- 4-5 times
- 6-7 times
- 8 plus times

1. On an average week how many times are you consuming alcohol?

- 0-1 times
- 2-3 times
- 4-5 times
- 6-7 times
- 8 times plus

1. If and when you consume alcohol, approximately how many drinks are you consuming per drinking episode?

- 1 drink
- 2-3 drinks
- 4-5 drinks
- 6-7 drinks
- 8 plus drinks

1. On an average night, how many hours of sleep do you get?

- 2-4
- 5-6
- 7-8
- 9-10
- 10+

1. Compared to your sleep prior to entering medical school, do you (approximately) sleep more, less, or the same amount?

- More
- Same amount
- Less

1. Do you currently use cigarettes?

- Yes
- No

1. If you answered yes to question five, please specify how many cigarettes you use per day.

- 1-2
- 3-4
- 5-6
- 7-8
- 8+

1. Do you currently use any vaporizing products?

- Yes
- No

1. If you answered yes to question seven, please specify which products you use.

- JUUL
- PAX
- Puff Bars
- Other e-cigarette/vaping products

1. Within the past three months, have you used recreational drugs?

- Yes
- No

1. Do you currently use any recreational drugs?

- Yes
- No

1. If you answered yes to question nine, please specify which drugs you use.

- Marijuana
- Cocaine
- Ecstasy (Molly)
- Heroin
- Methamphetamines
- Other

1. Within the past three months, have you used any prescription drugs that were not directly prescribed to you?

- Yes
- No

1. If you answered yes to question twelve, please specify which drugs you used.

- Adderall/Concerta
- Xanax
- Ambien
- Lexapro
- Valium
- Opioids
- Other

**Section 4: Perceptions About Medical School**

1. On a scale of 1-10 please rank how satisfied you were with your health prior to entering medical school - one being extremely dissatisfied, ten being extremely satisfied
2. On a scale of 1-10 please rank how satisfied you are currently with your health - one being extremely dissatisfied, ten being extremely satisfied
3. On a scale of 1-10 please rank how satisfied you were with your diet prior to entering medical school - one being extremely dissatisfied, ten being extremely satisfied
4. On a scale of 1-10 please rank how satisfied you are currently with your diet in medical school - one being extremely dissatisfied, ten being extremely satisfied
5. On a scale of 1-10 please rank your approximate stress level prior to entering medical school - one being no stress at all, ten being extremely stressed
6. On a scale of 1-10 please rank your approximate current stress level - one being no stress at all, ten being extremely stressed
